# Life-threatening arrhythmias due to faulty microdebrider during nasal sinus surgery

**DOI:** 10.4103/1658-354X.76468

**Published:** 2011

**Authors:** Rakesh Garg, Anjan Trikha, Preet M. Singh, P. K. Nishad

**Affiliations:** *Department of Anaesthesiology and Intensive Care, All India Institute of Medical Sciences, New Delhi, India*

Sir,

Success of surgical procedures is increasingly depending on modern highly sophisticated electrical equipments. Newer electrical gadgets with micro current delivery do not need a grounding electrode, which in case of malfunction can lead to life-threatening complications. We report such a life-threatening electric injury to the patient during functional endoscopic sinus surgery (FESS) with a faulty microdebrider.

A 25-year-old female was scheduled for functional endoscopic sinus surgery (FESS) for antrochoanal polyp antrochoanl polyp. Past medical history and physical examination were unremarkable. Her routine investigations were normal. Preoperatively, standard essential monitoring was set up and patient received standard balanced anesthesia [fentanyl (100 μg), propofol (120 mg) and vecuronium bromide (5 mg), oxygen, nitrous oxide, isoflurane and intermittent positive pressure ventilation].

After about 25 minutes, during surgery, when the power instrument, i.e., microdebrider (Medtronic Xomed XPS 3000, Jacksonville, FL, USA) was inserted in the sinus for polyp excision, the patient showed physical movement and heart rate suddenly increased from 92/minute to 160/minute. The saturation decreased from 98 to 70%. The surgical manipulation was stopped. Assuming inadequate neuromuscular blockade and analgesia, 1 mg of vecuronium and 25 μg of fentanyl was administered intravenously. The heart rate came back to 90/minute with an oxygen saturation of 98% within 10 seconds. The surgery was restarted. Immediately again, the heart rate increased to 178/minute and saturation decreased to 60% with dampening of pulse oximeter curve. Electrocardiogram rhythm was suggestive of ventricular tachycardia. It was noticed that the physical movement was only at left thoracic region and in the left arm. The surgeon was informed, surgery stopped and immediately the ventricular tachycardia spontaneously reverted to a sinus rhythm without the need of any intervention. The heart rate and oxygen saturation too became normal.

At this time, the surgeon informed that he could appreciate electric current in the microdebrider. The instrument was replaced and surgery was carried out uneventfully. At the end of surgery, the residual neuromuscular blockade was reversed and trachea extubated. Patient complained of pain and numbness in the fingers of left upper limb. On examining, it was revealed that there was superficial burn in the tips of index and middle fingers and erythyma at the thenar eminence of left arm. Postoperatively, an electrocardiogram and serum electrolytes were normal. The patient was observed for next 48 hours and had an uneventful recovery.

In the electrical devices, the hot and neutral leads connect to the device to power it, while the ground lead connects to the chassis of the device to return any current leaking from the device back to the ground. It is likely that in our case some defect in the hot wire and absence of grounding electrode led to delivery of current directly to the instrument chassis and thus to the patient.

Electrical injuries result from the flow of electric current through an inappropriate pathway. The extent of injury is determined by current flow and current density.[1] For current to flow through the body, there must be a difference of electrical potential between two points. The patient can provide an inadvertent ground connection through contact with instrument cases, beds etc. In our case, the left arm provided the ground as it was later realized to be in direct contact with metallic surface of the operating table. So the current flowed from nasal region to left arm via the thorax. In the thorax the current is split between the chest wall and the great vessels, which deliver the current directly to the myocardium.

The emergence of a microdebrider system has been useful for FESS because intraoperative blood loss has been reduced markedly.[2,3] Many of the electrical gadgets as electrocautery use a grounding plate, but the newer gadgets like microdebrider, bipolar and ophthalmic cautery lack such grounding electrodes. But on the other hand, these can be hazardous as it happened in our case with microdebrider.

Our patient had electric current shock with life-threatening arrhythmias from the faulty surgical instrument. At the first instance, we were misled that the change in hemodynamics was because of inadequate neuromuscular blockade and desaturation that occurred due to the movement of the arm leading to displacement of the pulse oximeter probe. But in the second instance, it was observed that the movement was only on specific portion of the body in spite of repeating the dose of neuromuscular blockade. This movement of left arm was due to direct stimulation of the muscles of left arm and shoulder. The current occurred due to faulty instrument, absence of grounding plate and some contact of the patient with the metallic operating table.

Subsequently, the instrument was checked by biomedical personnel in the postoperative period and was found to be electrically faulty and abnormal free flow of current was detected throughout the instrument. Current levels as low as 32 μA can cause hemodynamic collapse simulating ventricular tachycardia before ventricular fibrillation (VF) occurs.[4] Had we continued with this faulty microdebrider, the ventricular tachycardia could have changed to lethal cardiac rhythm.

We want to highlight that vigilance is required during use of electrical gadgets in the operating room. We suggest that with hand-held electric instruments delivering small amount of current and where grounding plates are not required like microdebrider, some type of earthing electrode should be present to prevent such complications. We should suspect electric fault in case of occurrence of arrhythmias or abnormal physical movement during the use of electrical gadget. Patients’ body should be protected from any contact with metallic surface during use of such electrical gadgets.
